# Helicobacter Pylori Serology in Relation to Hepatitis C Virus Infection and IL28B Single Nucleotide Polymorphism

**DOI:** 10.3390/jcm7030044

**Published:** 2018-03-05

**Authors:** Alexander Gutwerk, Thomas Wex, Kerstin Stein, Cosima Langner, Ali Canbay, Peter Malfertheiner, Alexander Link

**Affiliations:** 1 Department of Gastroenterology, Hepatology and Infectious Diseases, Otto-von-Guericke University Magdeburg, Leipziger Str. 44, 39120 Magdeburg, Germany; alexander@gutwerk.de (A.G.); t.wex@schenk-ansorge.de (T.W.); stein@hepatologie-magdeburg.de (K.S.); Cosima.Langner@med.ovgu.de (C.L.); ali.canbay@med.ovgu.de (A.C.); peter.malfertheiner@med.ovgu.de (P.M.); 2 Medical Laboratories for Clinical Chemistry, Microbiology and Infectious Diseases, Department of Molecular Genetics, 39124 Magdeburg, Germany

**Keywords:** *Helicobacter pylori*, hepatitis C virus, *IL28B*, rs12979860, CagA, serological rate, prevalence, cirrhosis, antibody

## Abstract

The aim of the study was to evaluate the serological rate of *Helicobacter pylori* (*H. pylori*) infection in patients with chronic hepatitis C virus (HCV) infection and determine any correlations with liver damage and *IL28B* single-nucleotide polymorphism (SNP). One hundred eighty-nine patients with chronic HCV infection were included in the study, and *H. pylori* status was defined based on anti-*H. pylori*-IgG or anti-CagA-IgG antibodies using enzyme-linked immunosorbent assay (ELISA). Liver damage was assessed using histology or transient elastography. *IL28B* C/T polymorphism (rs12979860) was evaluated in circulating blood cells using a PCR-based restriction fragment length polymorphism assay. Overall *H. pylori* serology was positive in 38.1% of our HCV-infected subjects. Among those, the anti-CagA-IgG positivity rate was 43.1% and was within the range of previously described populations of the same region. Highest prevalence of *H. pylori* was found in patients between 31 and 40 years compared to other age subgroups. The seropositivity rate was higher in the non-cirrhotic group than the cirrhotic one (45.4% vs. 20.0%, *p* < 0.05). No difference was found in *IL28B* genotype between *H. pylori*-positive and -negative cohorts. However, we observed a trend for the lower anti-CagA-IgG expression level in relation to the *IL28B* T-allele. Our results do not support an association between HCV and *H. pylori* infection. Whether *IL28B* SNP has a functional role in modulation of serological response to *H. pylori* CagA needs further investigation.

## 1. Introduction

Hepatitis C Virus (HCV) is a frequent infection, with up to 177 million people with present or past infections and an estimated global HCV prevalence around 2.5% [1]. HCV infection has reached up to a 3.5% prevalence in the Middle East, in North Africa, and in Eastern and Central Asia [2]. The prevalence in Germany is reported between 0.3 and 0.6%, which makes Germany a country with low prevalence within Europe [3,4]. Nevertheless, HCV infection is among the most important causes of chronic hepatitis, liver failure, and hepatocellular carcinoma (HCC) [5,6]. Furthermore, HCV has been considered a potential risk factor for intrahepatic cholangiocarcinoma and cancers of the pancreas, rectum, lung, and kidney [7,8].

*H. pylori* is an infectious disease that causes chronic gastritis and the majority of peptic ulcers [9,10]. More than 50% of the world´s population is infected with *H. pylori* [11]. Prevalence is by far higher in developing countries, but even within Europe a higher prevalence in Eastern Europe, compared to Western Europe, has been found [7,11]. For the region of Saxony-Anhalt, Wex et al. evaluated *H. pylori* seropositivity in a large cohort of patients showing a seroprevalence of 44.4% [12]. Since *H. pylori* infection is usually acquired during childhood, multiple studies have proposed the so-called “birth-cohort effect“, where the prevalence is higher in older groups and lower in young adults due to the decline in incidence of new infections [7,12,13].

Several studies have suggested that the effect of *H. pylori* infection is not limited to the stomach. For example, De Martel et al. [14] reviewed several studies that evaluated an association between *H. pylori* infection and biliary tract cancers. Among those, two studies found no *H. pylori* in the biopsies of patients and the control group. The remaining six studies reported a higher proportion of *H. pylori* positivity in patients compared to the controls, suggesting a positive association between *H. pylori* infection and biliary tract cancer. Moreover, there seems to be a link between *H. pylori* infection and HCC. Xuan et al. calculated a summary odds ratio of 13.6 for the association of *H. pylori* infection with the risk for HCC [15]. A close relative of *H. pylori*, *H. hepaticus* led to the development of HCC in an experimental infection model in mice [16]. With this animal model system of a *Helicobacter* species inducing hepatitis on the one hand and HCV being responsible to a large extent for HCC on the other hand, there is ongoing debate regarding the interaction of *H. pylori* and chronic HCV infections and its influence on progression to liver cirrhosis and HCC. In a meta-analysis, Wang et al. evaluated 12 studies looking for *H. pylori* (serological or PCR) in HCV-infected patients with a total amount of 1449 patients (547 patients from China and 549 from Italy) and 2377 control cases [17]. Compared to the controls, they found an odds ratio (OR) of 2.93 having a positive test for *H. pylori* in chronically infected HCV patients, no matter which state of HCV-related liver disease was present. In a subgroup analysis, the ORs were 4.48 for HCV-related cirrhosis and 5.45 for hepatocellular carcinoma [15]. The data, at least indirectly, implicate *H. pylori* infection as a risk factor for the progression of chronic HCV infection to liver cirrhosis and HCC.

The rs12979860 C/T single nucleotide polymorphism (SNP) is located 3 kb upstream of the *interleukin 28B* (*IL28B*) and is associated with spontaneous HCV clearance and HCV treatment success using historical pegINF and ribavirine-based therapy [18,19,20,21]. The presence of the T/T genotype is associated with a higher Ishak-score, suggesting that *IL28B* plays a role in the development of liver-fibrosis and -cirrhosis [19]. *IL28B* acts as an antiviral substance by regulating Treg cells and increasing adaptive cellular immunity stimulating genes through a Janus kinase (JAK) and Signal Transducer and Activator of Transcription (STAT) pathway [22,23]. Until now, no studies have evaluated the correlation between *IL28B* SNP and *H. pylori* infection.

Here, we evaluated the frequency of *H. pylori* infection in an HCV cohort with respect to liver cirrhosis in a tertiary medical center in Saxony-Anhalt in Germany. To estimate whether our results are within the range of other population cohorts in our region, we compared our data to recently published results. Furthermore, based on the known prognostic role of *IL28B* SNP in HCV treatment, *H. pylori* infection and seropositivity, for the first time, was correlated with *IL28B* polymorphism.

## 2. Materials and Methods

### 2.1. Study Design

From February 2011 to November 2015, we prospectively collected blood samples from HCV-infected patients treated in our outpatient unit of the Department of Gastroenterology, Hepatology and Infectious Diseases at Otto-von-Guericke University Magdeburg, Germany, for routine *IL28B* polymorphism analysis and evaluation of *H. pylori* status. All patients provided written informed consent for genetic analysis as part of the clinical routine testing. The study was evaluated by the local ethical committee and was given an exempt status for retrospective analysis of clinically obtained data.

### 2.2. Determination of HCV Status

Chronic HCV infection was evaluated by detecting anti-HCV antibodies either by the ECLIA test (anti-HCVII, Roche) or by quantitative RT-PCR using TaqMan 48 platform (Roche Diagnostics GmbH; Roche Applied Science, Mannheim, Germany). The detection limit (LOD) of this assay is 25 IU/mL serum. All analyses (including HBV and HIV diagnostics) were performed in the Central Clinical Laboratory of the Otto-von-Guericke University according to the manufacturer’s recommendation.

### 2.3. Determination of HCV IL28B SNP

Several different types of *IL28B* SNP have been used to predict the sustained virologic response (SVR) or rapid virologic response (RVR) in HCV patients. rs12979860 SNP is among the most frequently used, and routine testing of HCV patients has been implemented in our department in the past. Furthermore, this SNP has been shown to correlate with SVR not only in Genotype 1 but also in Genotypes 2 and 3 [24,25]. HCV *IL28B* rs12979860 C/T polymorphism has been identified by a PCR-based restriction fragment length polymorphism assay, which provides results that are consistent with other methods [19,26]. Here, extraction of human genomic DNA from whole blood was performed using DNA Mini Kit (Qiagen, Hilden, Germany) following the manufacturer’s recommendations. Amplification of DNA was done in a T3 Thermocycler machine (Biometra, Goettingen, Germany) with 15 μL of HotStar Taq Plus DNA Polymerase Mix (Qiagen, Hilden, Germany), 9.6 μL of RNase-free water, 0.2 μL each of forward (*IL28B*-F: 5′-gcttatcgcatacggctagg-3′; 50 μmol/L) and reverse primer (*IL28B*-R: 5′-aggctcagggtcaatcacag-3′; 50 μmol/L), and 5 μL of genomic DNA. The reactions were carried out as follows: enzyme activation at 95 °C for 15 min, 45 cycles of denaturation at 95 °C for 30 s, annealing at 60 °C for 30 s, extension at 72 °C for 1 min, followed by final extension at 72 °C for 10 min. After amplification of the 242 bp fragment, an enzymatic digestion was performed with Bsh 1236I restriction endonuclease (Fermentas, Waltham, MA, USA). Ten microliters of PCR product were digested with 0.4 µL of Bsh 1236I (10 U/μL) in 16.6 μL of RNase-free water and 3 µL of 10× Buffer R (Fermentas, Waltham, MA, USA) at 37 °C overnight. Restriction products were analyzed by agarose gel electrophoresis, ethidium bromide staining, and Hyperladder IV (Bioline, Luckenwalde, Germany) as a molecular weight marker. An E.A.S.Y RH system (Herolab, Wiesloch, Germany) was used for gel imaging. The digested fragments were 135 + 82 + 25 bp for the C-allele and 160 + 82 bp for the T-allele.

### 2.4. Determination of Liver Cirrhosis

The presence of liver cirrhosis was assessed by either histopathological report or was indirectly evaluated by studying the liver stiffness using transient elastography (FibroScan^®^, Echosens, Paris, France) [27,28].

### 2.5. Determination of H. pylori Status

Serological assessment of *H. pylori* status was performed as described previously [29]. Briefly, anti-*H. pylori-*IgG and anti-CagA antibodies were quantified using the *H. pylori*-IgG ELISA (Cat: 601040, BIOHIT, Helsinki, Finland) and the CagA-IgG kit (Cat: GD33, Genesis Diagnostics Ltd., London, UK) according to the manufacturer’s instructions, severally. Based on the presence of *H. pylori*-specific IgG (>30 EIU/mL) and/or the presence of anti-CagA IgG (>6.25 U/mL), patients were classified as *H. pylori*-positive. Patients lacking both antibodies (anti-*H. pylori* IgG and anti-CagA IgG) were considered *H. pylori*-negative.

### 2.6. Comparison of the Data to the Previously Reported Cohorts

To compare the results with previously available *H. pylori* seroprevalence from our region, we used two recently published cohorts. Wex et al. [12] have prospectively evaluated the *H. pylori* seropositivity in patients visiting our emergency department. Franck et al. [30] evaluated the *H. pylori* seropositivity in consecutive blood donors also in the same region. All studies used similar criteria for defining the *H. pylori* seropositivity and similar kits (described above) to avoid any methodological bias. For detailed information, we kindly refer to the original publications [12,30].

### 2.7. Statistical Analysis

Data were entered into a database using Excel (Microsoft Office Package, Redmond, WA, USA). Age is shown as mean and standard deviations (mean ± SD), and was analyzed by an unpaired Student’s *t*-test. Categorical data (e.g., gender, *H. pylori* status) are presented as frequencies; comparisons were performed using the chi-square or Fisher´s exact test. Differences in *IL28B* and *H. pylori* infection were compared by a Mann–Whitney test using two groups or a Kruskal–Wallis test for more than two groups. All tests were applied two-sided with a significance level of *p* < 0.05.

## 3. Results

Patients’ characteristics are presented in Table 1. Briefly, 105 out of 189 patients were males (55.6%), and 84 were females (44.4%). The mean age was 51.3 years ranging from 19 to 93 years. With respect to the origin of patients, Western and Eastern Europe were the main regions, *n* = 131/69.3% and 45/23.8%, respectively. Few patients originated from Asia (*n* = 8) or other regions (*n* = 5). HCV genotype data were available for 182 patients with the majority of patients with Genotype 1 *n* = 156 (85.6%). The remaining patients had Genotype 2 *n* = 4 (2.2%), Genotype 3 *n* = 18 (9.9%), Genotype 4 *n* = 3 (1.6%), and Genotype 6 *n* = 1 (0.5%).

The overall serological rate of *H. pylori* antibodies in our cohort was 38.1%, without any difference between males and females (Table 1). Ethnicity was associated with a higher *H. pylori* detection rate for Eastern Europe compared to Western countries (64.4% vs. 29%, *p* < 0.001). Patients co-infected with HBV demonstrated a significantly higher rate of *H. pylori* seropositivity compared to those without coinfection. Furthermore, patients with Genotypes 3 and 4 compared with those with Genotype 1, showed higher *H. pylori* seropositivity. Interestingly, patients with cirrhosis had a significantly lower *H. pylori* seropositivity rate than those without cirrhosis (Table 1); this was in spite of the older age of cirrhotic groups, compared to non-cirrhotic groups (60.6 vs. 47.5 years).

**Figure 1:**
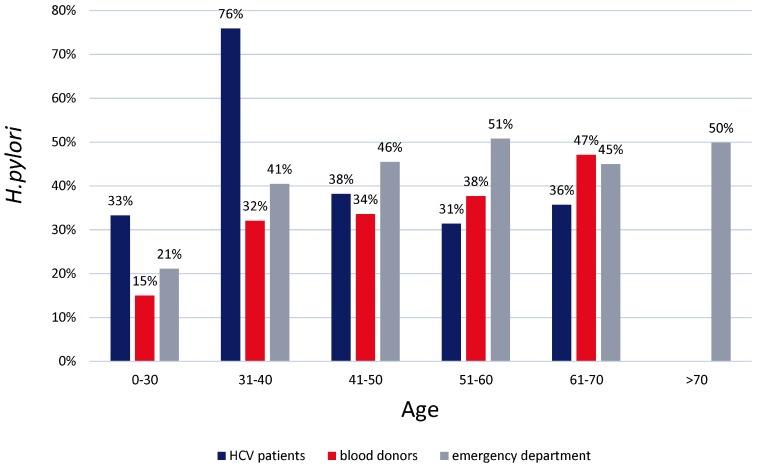
Distribution of *H. pylori* infection in our cohort in comparison to the general population (subjects in emergency department [12] and healthy blood donors [30]) estimated using two independent cohorts.

*H. pylori* seropositivity showed no birth-cohort effect compared to similar population-based studies (Figure 1). The positive *H. pylori* status was found to be significantly higher for patients from 31 to 40 years, whereas younger and older subgroups revealed similar rates between 31% and 39%. Compared to two other studies from the same local area of Saxony-Anhalt, the serological *H. pylori* positivity rate in the HCV cohort was lower than that in the general population, but higher than those identified in healthy blood donors [12,30]. In comparison to previously published results, we were unable to observe a typical age-related “birth-cohort effect”, suggesting that, in the HCV cohort, additional factors likely included a typical distribution with an increasing *H. pylori* positivity in older patients (Table 2).

### H. pylori Infection and IL28B Genotype

**Figure 2:**
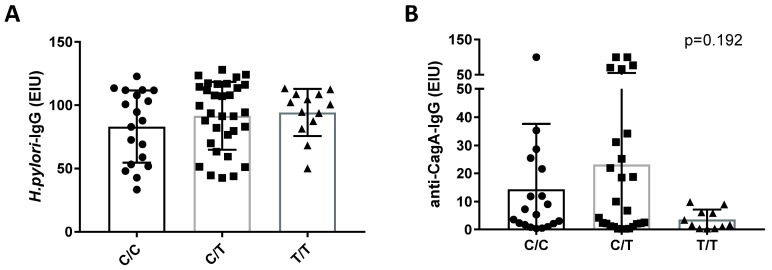

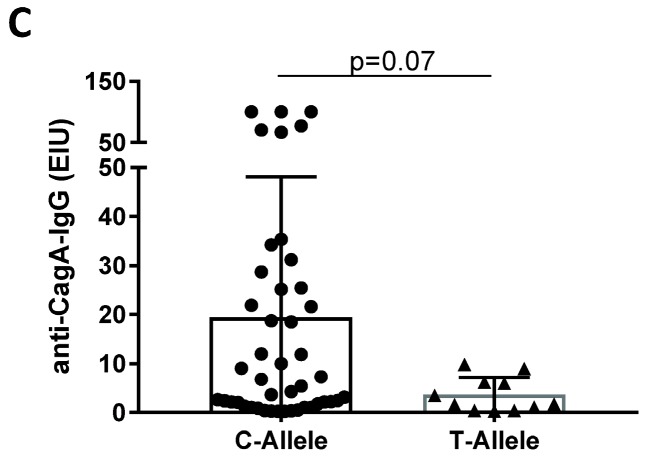
*H. pylori* and anti-CagA-seropositivity in relation to *IL28B* SNP. (**A**) Anti-*H. pylori*-IgG in *IL28B* C/C, C/T and T/T genotypes. (**B**) Anti-CagA-IgG in IL28B C/C, C/T, and T/T genotypes. (**C**) Anti-CagA-IgG in *IL28*B C- and T-alleles. Two groups were compared using a Mann–Whitney test and more than two groups using a Kruskal–Wallis multiple comparison test.

The role of *IL28B* in *H. pylori* infection had not been previously studied. We questioned whether patients with different *IL28B* genotypes have different immune responses during *H. pylori* infection. Here, we evaluated the serological anti-*H. pylori*-IgG and anti-CagA-IgG from *H. pylori*-infected subjects in relation to different *IL28B* genotypes. As shown in Table 1 and Figure 2, there was no significant difference in *H. pylori* seropositivity in the different *IL28B* genotypes, suggesting a similar distribution of *IL28B* polymorphisms among *H. pylori*-positive and -negative cohorts. In a recent study, we showed that CagA-seropositivity may be dependent on various factors and hypothesized the role of genetics [29]. Therefore, we evaluated whether *IL28B* SNP has any association with *H. pylori*/CagA-seropositivity. No difference was found in anti-*H. pylori*-IgG levels. However, there was a remarkable trend for lower anti-CagA-IgG levels in patients with the *IL28B* SNP genotype, which was true for both T/T (*p* = 0.113) and T-allele (*p* = 0.077) in comparison to patients with C/C and C/T genotypes or C-allele, respectively (Figure 2). Since our cohort of HCV-infected subjects included HBV and HIV-coinfected patients, we questioned whether co-infection with HIV in particular could lead to lower antibody levels. The analysis of *IL28B* SNP revealed, however, that all HIV co-infected subjects had either a C/C or a C/T genotype and therefore showed no association with lower anti-CagA-IgG. Furthermore, the proportion of HBV-co-infected subjects, based on the *IL28B* SNP, was similar to that of the total HCV cohort, with C/C 30%, C/T 60%, and T/T 10% compared to the total HCV cohort with C/C 28%, C/T 54%, and T/T 19%. Therefore, at least based on our data we did not observe any correlation between HBV and HIV co-infection and lower anti-CagA-IgG with relation to *IL28B* SNP.

## 4. Discussion

This work provides an up-to-date view on the prevalence of *H. pylori* infection in HCV-infected subjects and provides initial data for a potential correlation between *IL28B* polymorphisms and CagA-seropositivity. We show that patients in the HCV cohort have similar *H. pylori* positivity as the general population in the same region; however, the presence of liver cirrhosis was associated with a lower *H. pylori* positivity rate.

Overall *H. pylori* positivity rate of about 38% in the HCV cohort fits with epidemiological data from other European countries [32], in particular with the studies from Germany [33,34] and with the region of Saxony-Anhalt [12,30]. The high serological *H. pylori* rate in younger patients, in particular those between 30 and 40 years, corresponds to other studies showing an elevation of *H. pylori* seroprevalence in HCV patients, for example [17]. However, in contrast, our data does not provide any evidence for potential interaction between either infection since *H. pylori* and HCV infections are acquired at different ages. In our study cohort, approximately 50% of all patients positive for *H. pylori* in the group of 31–40 years were immigrants from Eastern Europe. Therefore, the rather high *H pylori* rate in this group (31–40 years) may be driven by the inclusion of HCV patients from areas with high *H. pylori* prevalence, with potential differences in transmission [35,36,37,38,39].

Interestingly, in contrast to previous studies from our region [12,30], we observed a missing “birth-cohort effect” in HCV-infected subjects; this observation implies additional factors or potential selection bias. The subjects from our cohort were recruited from a tertiary center where the majority of patients underwent specialized medical care. This includes regular visits, especially in the era of interferon-based therapy. In particular, those patients with liver fibrosis/cirrhosis were very likely to undergo additional diagnostics, including upper GI-endoscopy on a routine basis for a screening of varices during long-term follow-up. The routine workup in Europe is to perform histology, which substantially increases the chance of *H. pylori* detection, and eradication was accordingly offered to everyone even though the majority of patients were probably asymptomatic [40,41]. However, even regular contact with a gastroenterologist/hepatologist makes it more likely that *H. pylori* infection is detected by chance or with mild symptoms. Perez-Perez et al. discussed this aspect in a study where a lower serological *H. pylori* rate was also identified frequently in patients with regular contact to medical practitioners [42]. Our data support this conclusion although the clinically relevant meaning of the data has yet to be obtained. Notably, the majority of guidelines with a scope to cirrhosis and portal hypertension recommend screening for esophageal varices by EGD [43]. Therefore, this strategy may increase the likelihood of detecting *H. pylori* infection with subsequent eradication therapy leading to lower *H. pylori* infection rates in patients with cirrhosis. In addition, as patients suffering from liver cirrhosis were older than patients without cirrhosis, we assume that the intensive medical care due to advanced liver damage (cirrhosis) might lead to lower *H. pylori* rates in elderly patients, whereas the inclusion of patients from countries with a high prevalence of *H*. *pylori* led to higher *H. pylori* rates in younger patients. Together, this might explain the unusual age-related pattern of *H. pylori* seropositivity. We speculate that testing for *H. pylori* infection in addition to HCV testing would provide an additional long-term health benefit for patients, reducing the chance of developing preneoplastic conditions or complications such as peptic ulcer or gastric cancer.

*IL28B* SNP is well known to influence spontaneous and treatment-induced clearance of HCV infection [19,20]. In this work, a possible association between *IL28B* SNP and *H. pylori* was evaluated for the first time. First, we found no difference in *IL28B* genotypes among the *H. pylori*-positive and *H. pylori*-negative groups, which indirectly confirms the validity of the groups. Second, in our previous work, we found that only around 32.3% of subjects that have *H. pylori* infection and *H. pylori* CagA positive strains have a positive serology [29]. Although we found crucial evidence for the role of the *vacA* genotype as an important determinant for seropositivity, we also questioned whether the presence of potential genetic host factors could have an influence on the serological profile. Because of the pronounced role of *IL28B* in the immune response to HCV infection, we further questioned whether *IL28B* could be involved in *H. pylori* infection. The association analysis of the *IL28B* SNP genotype revealed a trend for the lower anti-CagA-IgG in subjects with T/T-genotype and T-allele; in other words, none of the subjects with a T/T genotype had high anti-CagA-IgG levels. As discussed, there may be multiple factors such as genetic polymorphism—such as HLA or, in our case, *IL28B*—which are likely to be associated with immune response on *H. pylori* and therefore serological conversion [44]. Furthermore, it is reported that the *IL28B* C/C genotype is generally more frequent in subjects in East Asia [19] and that Asian countries have a rather high anti-CagA-seropositivity frequency compared to other countries [29]. At this stage, we may only speculate that C- or T-alleles of *IL28B* SNP could be involved in the modulation of immunological response to the CagA virulence factor. However, it needs to be mentioned that the number of *H. pylori* positive patients with *IL28B* T/T genotype was quite small and that further studies are needed.

There are several limitations that need to be mentioned. First of all, the number of samples is relatively small, which is at least partly related to the low HCV infection prevalence from one side and to changes in therapeutic management with the availability of new effective drugs that make *IL28B* testing unnecessary. Because of the limited sample size, we did not perform any additional subgroup analysis to evaluate an association between HCV and *H. pylori*. As we show the lower *H. pylori* seropositivity in cirrhotic patients, we believe that this could be related to prior eradication treatments. Unfortunately, because of the retrospective data analysis, we could not obtain data regarding former eradications/visits. The main novelty of the work, however, is related to the *IL28B* SNP and *H. pylori* analysis. Nevertheless, we only implemented rs12979860 SNP, which shows a good correlation to sustained viral response. Other types of SNP of the *IL28B* gene may be important as well and need to be considered in the future [24,25]. Finally, we provide the data to *IL28B* SNP in HCV-infected subjects. Whether a similar immunological pattern or correlation can be found in HCV-negative cohorts, especially taking into account the origin of the patients (European vs. Asian), needs to be addressed in the future. With the availability of highly effective anti-HCV drugs, however, the question regarding the hypothetical interaction/association between *H. pylori* and HCV may be more difficult to address.

## 5. Conclusions

We demonstrate that the serological rate of *H. pylori* in patients with chronic HCV was within the range of seropositivity previously described for the same region. The highest *H. pylori* rate was found in young adults, while the lowest rate was found in patients with liver cirrhosis. Analysis of the *IL28B* rs12979860 polymorphism revealed a trend for the lower anti-CagA-IgG seropositivity related to T-alleles. Whether *IL28B* SNP may be associated with host-dependent modulation of serological response to *H. pylori* infection needs to be confirmed in the future. Furthermore, further evaluation is required to determine whether *H. pylori* infection may be associated with worse outcome or prognosis in HCV patients and whether *H. pylori* eradication may provide an objectively measurable long-term benefit to HCV infected subjects in addition to healthy gastric mucosa.

## Figures and Tables

**Table 1:** Serological rate (%) of *H. pylori* infection stratified by sex, ethnicity, other co-infections, and the presence of liver cirrhosis.

Characteristic	Total	*H. pylori*-Positive	*H. pylori*-Negative	*p*-Value
	*n*	*n* (%)	*n* (%)	
HCV-positive	189 *	72 (38.1%)	117 (61.9%)	n.s.
Gender				
male	105	39 (37.1%)	66 (62.9%)	
female	84	33 (39.3%)	51 (60.7%)	
Age	51.32 ± 13.4	47.11 ± 13.4	53.91 ± 13.3	<0.001 ^#^
≤40 years	44	27 (61.4%)	17 (38.6%)	<0.001
>40 years	145	45 (31.0%)	100 (69.0%)	
HCV genotype	182 *	71 (39.1%)	111 (60.9%)	
1	156	55 (35.3%)	101 (64.7%)	
2	4	1 (25%)	3 (75%)	
3	18	13 (72.2%)	5 (27.8%)	
4	3	2 (66.7%)	1 (33.3%)	
5	0	0	0	
6	1	0	1 (100%)	
Origin:	176 *			
Western Europe	131	38 (29.0%)	93 (71.0%)	<0.001
Eastern Europe	45	29 (64.4%)	16 (35.6%)	
Coinfection to HCV:	188 *			
None	158	53 (33.5%)	105 (66.5%)	<0.005
HBV	22	15 (68.2%)	7 (31.8%)	
HIV	8	3 (37.5%)	5 (62.5%)	
Liver status:	189 *			
no liver cirrhosis	134	61 (45.5%)	73 (54.5%)	<0.005
liver cirrhosis	55	11 (20.0%)	44 (80.0%)	
*IL28B*-SNP genotype	169 *			
C/C	45	19 (42.2%)	26 (57.8%)	n.s.
C/T	91	32 (35.2%)	59 (64.8%)	
T/T	33	13 (39.4%)	20 (60.6%)	

Data shown as the absolute number with % or as the mean ± SD. * retrospectively not all data were available systematically for all subjects. Analyses were done with chi-square or Fisher’s exact tests with the exception of ^#^ where an unpaired *t*-test was used; n.s. = not significant. HBV infection is defined by positive HBs-Ag and/or HBV-DNA to exclude past HBV infection.

**Table 2:** Serological rate of *H. pylori* infection in the HCV cohort stratified by age.

Age in Years	No. of *H. pylori*-Positive Subjects/All Subjects (%)		
	Current Study	Wex et al. 2011	Franck et al. 2017
overall	72/189 (38.1%)	1029/2318 (44.4%)	149/516 (28.9%)
≤30	5/15 (33.3%)	61/289 (21.1%)	15%
31–40	22/29 (75.9%)	75/185 (40.5%)	32%
41–50	13/34 (38.2%)	122/268 (45.5%)	34%
51–60	22/70 (31.4%)	167/329 (50.8%)	38%
61–70	10/28 (35.7%)	167/371 (45.0%)	47%
>70	0/13 (0%)	437/876 (49.9%)	n.a. *

Relative proportion of *H. pylori* seropositivity in subjects presented in emergency department (Wex et al. 2011) [12] or in blood donors (Franck et al. 2017) [31]. *H. pylori* positivity status was defined in all three studies using similar criteria: anti-*H. pylori*-IgG+ and/or anti-CagA-IgG+. n.a.* age of >70 years was an exclusion criteria for blood donation and therefore not available.
